# All-LCP Terahertz Metasensor with Dual Quasi-BIC Resonances for Dual-Range Refractive Index Sensing

**DOI:** 10.3390/bios16040221

**Published:** 2026-04-15

**Authors:** Yan Zhang, Mengya Pan, Qiankai Hong, Shengyuan Shen, Conghui Guo, Yaping Li, Yanpeng Shi, Yifei Zhang

**Affiliations:** 1 School of Integrated Circuits, Shandong University, Jinan 250100, China; 2 Shandong Key Laboratory of Metamaterial and Electromagnetic Manipulation Technology, Jinan 250100, China

**Keywords:** terahertz metasensor, all-LCP metasurface, dual quasi-BIC resonances, refractive-index sensing, differential readout, biosensing

## Abstract

Terahertz (THz) metasurface biosensors still encounter difficulties in simultaneously achieving high spectral resolution and stable readout across different refractive-index regimes. In this work, an all-liquid-crystal-polymer (LCP) THz metasensor supporting dual quasi-bound states in the continuum (quasi-BIC) resonances is proposed for regime-dependent refractive-index sensing. By introducing structural asymmetry into a periodic LCP cubic-cluster metasurface, two pronounced resonances are generated with quality factors (Q factors) of 6811 and 2526, respectively. Near-field distributions and multipole decomposition analysis indicate that the two resonances possess distinct electromagnetic features, which result in different responses to surrounding dielectric perturbations. In the low-refractive-index range of 1.0–1.5, the two resonance frequencies exhibit a linear variation with refractive index, yielding sensitivities of 122 GHz/RIU and 179 GHz/RIU, respectively. These dual-mode linear responses further offer a foundation for concentration- and temperature-related evaluation through analyte refractive-index mapping. In the higher-refractive-index range of 1.5–1.8, the intermodal frequency difference shows improved linearity with refractive index compared with the individual resonance frequencies, enabling a differential readout scheme with enhanced robustness against common perturbations. The results demonstrate that the proposed all-LCP dual-quasi-BIC metasensor not only enables high-resolution THz refractive-index sensing, but also establishes a regime-dependent spectral readout approach for different dielectric-response intervals.

## 1. Introduction

Terahertz (THz) technology has emerged as an effective analytical method in biochemical research because it enables label-free, non-ionizing, and non-destructive probing of analytes through their dielectric responses and intermolecular interactions. These properties have made THz spectroscopy attractive for biological sensing [1], liquid analysis [2], molecular identification [3,4], and disease-related diagnostics [5,6]. At the same time, modern medicine is increasingly driven by the need for precision diagnostics, dynamic disease monitoring, and minimally invasive testing. In this broader context, liquid biopsy and related photonic biosensing platforms have attracted considerable attention because they offer new possibilities for rapid, sensitive, and clinically adaptable analysis of biomarkers in accessible biofluids [7,8]. Within this evolving technological landscape, THz sensing can provide a complementary route for label-free biochemical analysis, especially when spectral response can be enhanced and read out with sufficient resolution and robustness.

A central challenge in THz sensing is the weak interaction between THz waves and trace analytes due to the large wavelength mismatch [9]. To address this limitation, metasurfaces have been widely used to enhance subwavelength field confinement and improve spectral readout capability. Early THz sensing studies mainly relied on metallic resonant metastructures, which established the fundamental route for local-field enhancement and resonance-based spectral readout [2,3]. However, the inherent Ohmic loss of metals tends to broaden resonance linewidths, thereby limiting further enhancement in spectral sharpness and sensing resolution [4,5]. To address this limitation, increasing attention has been directed toward all-dielectric metasurfaces, in which low-loss dielectric resonators can support Mie-type modes with strong field confinement and high spectral selectivity [10,11]. Representative studies have further indicated that all-dielectric THz metasurfaces can support cytokine detection [12], molecular fingerprint identification [13], low-concentration biomolecular sensing [14], and liquid-sample sensing [15] with improved spectral characteristics. Among the dielectric materials explored for THz devices, liquid crystal polymer (LCP) has attracted particular attention because of its low loss, low water absorption, chemical stability, mechanical flexibility, and compatibility with lamination, packaging, and compact integration from microwave to THz frequencies [16,17]. These developments suggest that LCP-based all-dielectric metasurfaces provide a promising platform for low-loss, compact, and practically implementable THz sensing devices.

Among various resonance-engineering mechanisms, bound states in the continuum (BICs) and quasi-bound states in the continuum (quasi-BICs) have received considerable attention for high-performance sensing applications. Ideal BIC modes are fully decoupled from free-space radiation and thus cannot be directly observed in conventional far-field spectra. When symmetry breaking or structural perturbation is introduced, an ideal BIC can be converted into a quasi-BIC with finite radiation leakage and an ultrahigh quality factor (Q factor), which is advantageous for detecting weak environmental changes [18,19]. On this basis, BIC-inspired metasurfaces have been widely investigated for refractive-index sensing [20,21], temperature sensing [22], differential sensing [23], molecular detection [24,25], and fingerprint enhancement [26,27]. Recent studies have further demonstrated the potential of quasi-BIC-enabled terahertz sensing. For example, Lou et al. developed a calibration-free terahertz ultrafast metasurface for monitoring gastric cancer progression, in which time-delayed transmission spectra were normalized to suppress environmental errors and improve sensing robustness [28]. Wang et al. reported a portable quasi-BIC terahertz metasensor for ultrasensitive biodetection of low-concentration analytes, highlighting the utility of sharp quasi-BIC resonances in compact biochemical sensing [29]. These studies confirm the value of quasi-BIC resonances as an effective route toward high-resolution THz sensing. However, existing studies remain mainly focused on sensitivity enhancement or single-parameter refractive-index sensing [30,31,32,33]. In practical analyte systems, dielectric responses may vary across different refractive-index intervals, and concentration- and temperature-related perturbations are often coupled through refractive-index changes. Therefore, beyond obtaining high-Q resonances, it remains important to develop THz metasensors that support flexible spectral observables and analyte-related readout strategies under different dielectric-response regimes.

In this work, an all-LCP THz metasensor composed of periodic cubic-cluster resonators is presented, in which dual quasi-BIC resonances are induced through structural symmetry breaking. The physical origin of the symmetry-protected state and the formation mechanisms of the two resonant modes are clarified by analyzing spectral responses, near-field distributions, and multipole decomposition. Based on this, a regime-dependent sensing framework is developed. In the lower refractive-index range, the two resonances are utilized as dual spectral observables for refractive-index readout, as well as for concentration- and temperature-related analysis through analyte refractive-index mapping. In the higher refractive-index range, the intermodal frequency difference is used as a more stable observable to achieve differential readout. The influence of analyte thickness is further investigated to assess surface sensitivity and near-field penetration behavior. By integrating the spectral advantages of dual quasi-BIC resonances with the low-loss and integration-compatible features of LCP, a compact THz sensing platform is provided, offering a practical approach for high-resolution and application-oriented biochemical sensing.

## 2. Materials and Methods

**Figure 1:**
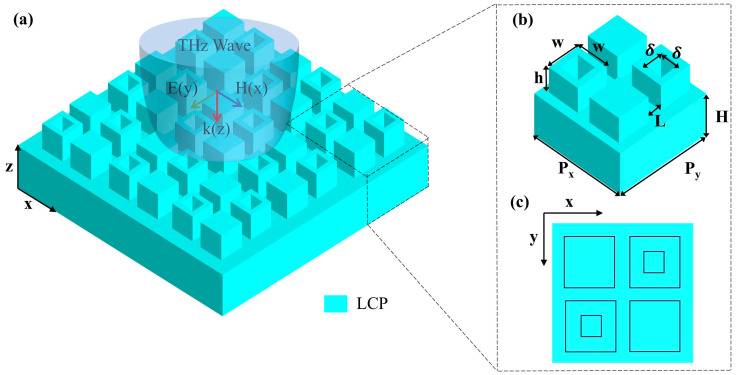
(**a**) Schematic of the all-dielectric metasurface composed of periodic LCP cubic clusters. (**b**) Unit cell of the periodic structure. (**c**) Top view of the unit cell, showing the arrangement of solid and nested square cubic subunits.

The proposed all-LCP dielectric metasurface is shown in Figure 1a and consists of a periodic tetramer array fabricated on an LCP platform. Each unit cell contains four LCP cubic resonators arranged in a square lattice, where two diagonally positioned cubes are perforated with central square cavities, while the remaining two are kept solid. This hybrid tetramer configuration serves as the structural basis for symmetry modulation and enables the subsequent excitation of quasi-BICs. The geometrical parameters of the metasurface are defined in Figure 1b,c. The lattice constants are Px = Py = 400 μm, the side length of each cubic resonator is L = 50 μm, the total thickness of the LCP substrate is H = 35 μm, and the height of the upper structured layer is h = 15 μm. The refractive index of LCP is set to n = 1.8 [16], and the surrounding medium is air. In the present model, LCP is approximated as a low-loss dielectric with a constant refractive index [16,17,34], so that the analysis focuses on the structural resonance behavior of the proposed metasurface. The patterned region is supported by the same LCP layer rather than by an additional substrate of a different material. In principle, the proposed all-LCP metasurface could be realized by femtosecond-laser micromachining of the LCP layer to define the patterned tetramer structure. The transmission spectra are calculated within the frequency ranges of 0.8–0.9 THz and 1.125–1.225 THz, which cover the resonant modes of interest.

To quantify the structural asymmetry of the tetramer unit, an asymmetry parameter δ is introduced and defined as the side length of the square cavity etched into the two diagonal resonators. The electromagnetic response of the metasurface is numerically analyzed using Ansys HFSS 2023 R1. In the simulations, a single unit cell is employed to represent the infinite periodic array. Periodic boundary conditions are applied along the x- and y-directions, while Floquet ports are applied along the z-direction to implement the normally incident plane-wave excitation and to obtain the corresponding transmission response. The structure is excited by a normally incident transverse magnetic (TM) wave propagating along the z-direction. This excitation is adopted as a representative configuration, since its in-plane electric field can effectively couple to the symmetry-broken tetramer unit and enable the excitation of the quasi-BIC modes analyzed in this work. For the sensing simulations, the analyte is modeled as a uniform dielectric overlayer covering the metasurface, with a default thickness of t = 20 μm. This value is adopted as a representative baseline thickness for the main sensing analysis [20], providing a clear sensing response under a finite analyte loading condition, while the influence of analyte thickness is examined separately in the later thickness-dependent study. Based on this simulation model, the transmission spectra and field distributions are utilized in subsequent analyses of modal evolution, refractive-index sensing, concentration- and temperature-related response, and thickness-dependent behavior. From a practical perspective, the proposed all-LCP metasurface benefits from the low water absorption, low dielectric loss, and good chemical stability of LCP in THz applications. As in other high-Q resonant systems, fabrication tolerances and environmental variations, such as temperature and humidity, can lead to resonance shifts, while the intrinsic material loss of LCP imposes a limit on the achievable Q factor. Nevertheless, these effects do not alter the suitability of LCP as a low-loss platform for THz sensing under practical conditions [34]. This type of refractive-index-perturbation treatment is also commonly adopted as an initial sensing model in related THz metasurface studies, where the sensing medium is first represented by an effective surrounding refractive index or background dielectric environment before more realistic loss effects are considered in greater detail [19,35,36].

## 3. Results

**Figure 2:**
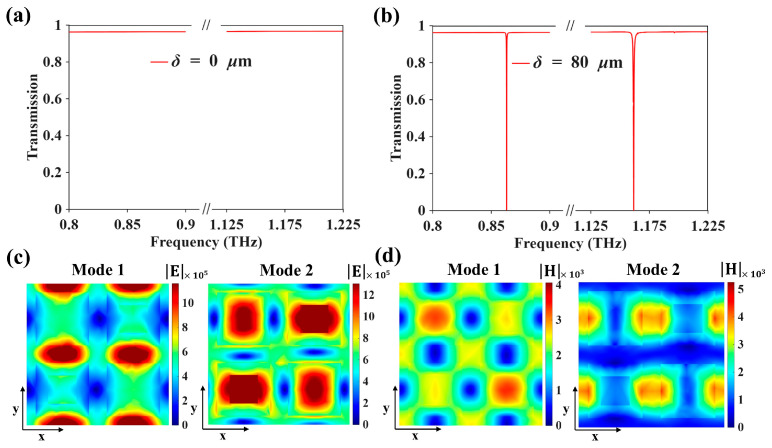
(**a**) Transmission spectra of the metasurface with perfect structural symmetry (δ = 0 μm). (**b**) Transmission spectra after introducing structural asymmetry (δ = 80 μm), where two quasi-BIC resonances (Mode 1 and Mode 2) appear. (**c**) Electric field (|E|) distributions of Mode 1 and Mode 2 at the resonance frequencies. (**d**) Magnetic field (|H|) distributions of Mode 1 and Mode 2 at the resonance frequencies.

Figure 2 presents the transmission spectra and the corresponding near-field distributions of the proposed metasurface under different symmetry conditions. When the asymmetry parameter is set to δ = 0 μm, the tetramer unit remains fully symmetric, and no evident resonance dip appears within the investigated spectral windows, as shown in Figure 2a. This behavior agrees with the presence of symmetry-protected dark states, which remain effectively decoupled from the external radiation channel in the symmetric configuration [29]. After introducing structural asymmetry by setting δ = 80 μm, two distinct resonances emerge in the transmission spectrum, as illustrated in Figure 2b. The Q factors reported in this work are calculated from the simulated transmission spectra using $Q=f_{0}/\Delta f$ [30], where $f_{0}$ is the resonance frequency and Δf is the corresponding resonance linewidth. These resonances are located at 0.863 THz and 1.162 THz, denoted as Mode 1 and Mode 2, respectively, with extracted Q factors of 6811 and 2526. The appearance of these narrow spectral features indicates that symmetry breaking lifts the protection of the original dark states and enables finite radiative coupling, thereby converting them into observable quasi-BIC resonances [31,32]. The electric- and magnetic-field distributions at the two resonance frequencies are shown in Figure 2c,d. For Mode 1, the electric field is primarily concentrated in the gaps between adjacent resonators, indicating relatively strong inter-resonator coupling. In contrast, Mode 2 exhibits a more internally confined field distribution within the tetramer region. The magnetic-field patterns also show clear differences between the two resonances. These observations indicate that Mode 1 and Mode 2 exhibit different modal characteristics in the symmetry-broken tetramer structure. In particular, the stronger gap-field confinement of Mode 1 is consistent with reduced radiative leakage and thus a higher Q factor, whereas the modal distribution of Mode 2 leads to a greater field exposure to the surrounding dielectric, resulting in a larger sensing response.

**Figure 3:**
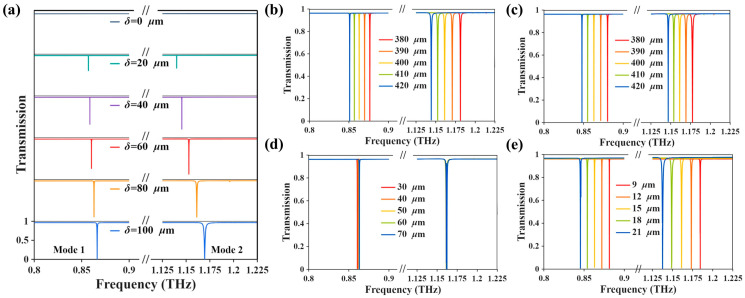
(**a**) Evolution of the transmission spectra with the asymmetry parameter δ. (**b**) Transmission spectra as a function of Px. (**c**) Transmission spectra as a function of Py. (**d**) Transmission spectra as a function of L. (**e**) Transmission spectra as a function of h.

The asymmetry-dependent transmission spectra shown in Figure 3a provide further evidence for the quasi-BIC interpretation. As δ increases from 0 to 100 μm, the two resonances gradually emerge from the symmetry-protected limit and show progressive linewidth broadening, which is consistent with the expected quasi-BIC evolution induced by symmetry breaking. Figure 3b–e further summarizes the spectral evolution obtained by scanning the remaining key geometrical parameters Px, Py, L and h. The results show that changes in Px, Py and h primarily lead to noticeable shifts in the resonance frequencies, thereby determining the spectral positions of the two modes. In contrast, variation of L mainly affects the resonance linewidth and thus plays a dominant role in tuning the Q factor, with relatively minor influence on the resonance frequencies. Based on these trends, the final values Px = Py = 400 μm, L = 50 μm and h = 15 μm were selected as a balanced working configuration, taking into account suitable spectral positioning, relatively high Q values, clear dual-resonance formation, and practical structural feasibility.

To further clarify the physical origin of the two resonances, the scattering response of the metasurface was examined using Cartesian multipole decomposition, including the electric dipole (ED), toroidal dipole (TD), magnetic dipole (MD), electric quadrupole (EQ), and magnetic quadrupole (MQ) contributions. The multipole post-processing was performed using the open-source MATLAB R2025a implementation MENP [37].

**Figure 4:**
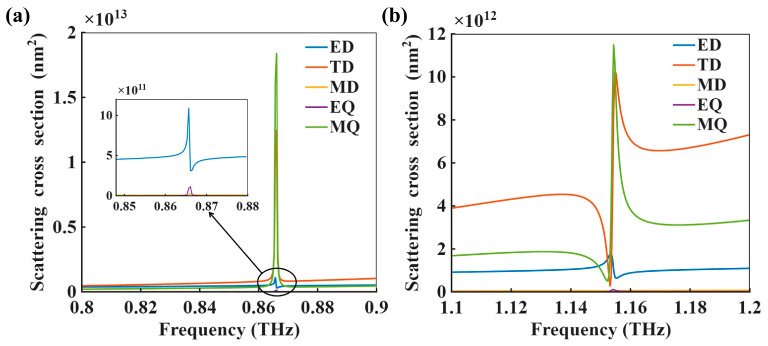
Multipole decomposition of the scattering cross sections. (**a**) Contributions of the electric dipole (ED), toroidal dipole (TD), magnetic dipole (MD), electric quadrupole (EQ), and magnetic quadrupole (MQ) near the resonance of Mode 1. The inset shows the enlarged region around the resonance frequency. (**b**) Multipole contributions near the resonance of Mode 2.

As shown in Figure 4a, the resonance of Mode 1 is mainly dominated by the MQ contribution, while the TD term also provides a noticeable contribution near the resonance frequency. In comparison, the ED, MD, and EQ components remain relatively weak within the same spectral region. For Mode 2 in Figure 4b, the scattering response is also dominated by the MQ and TD terms, but the hybridization between these two multipolar components is more significant than that observed for Mode 1. These results indicate that both resonances originate from higher-order multipolar states rather than conventional dipolar resonances. Moreover, the overall MQ–TD response of Mode 1 is stronger than that of Mode 2, which is consistent with its more pronounced trapped-mode character and higher Q factor. By contrast, although Mode 2 is also dominated by MQ and TD contributions, their relative dominance is less pronounced and the overall response amplitude is lower, which is consistent with a less confined resonant character, leading to a lower Q factor and a stronger sensitivity to refractive-index perturbations.

A comparison of the proposed metasurface with representative all-dielectric THz sensing platforms is summarized in Table 1. As shown in Table 1, the present structure does not aim to maximize a single performance metric such as sensitivity alone. Instead, it combines a relatively high-Q dual-resonance response with an all-LCP material platform and a regime-dependent readout strategy. In particular, the proposed sensor exhibits a relatively high-Q dual-resonance response while maintaining competitive sensing capability. More importantly, the use of two quasi-BIC resonances enables flexible observable selection in different refractive-index intervals, which is not emphasized in the compared designs. Here, the figure of merit (FOM) is defined as $FOM=S/\Delta f=S\times Q/f_{0}$ [38], where S is the sensitivity, Δf is the full width at half maximum (FWHM), $f_{0}$ is the resonance frequency. In this sense, the proposed metasurface should be regarded as a practically oriented and adaptively readable THz sensing architecture rather than a design optimized only for the highest Q factor or sensitivity.

**Figure 5:**
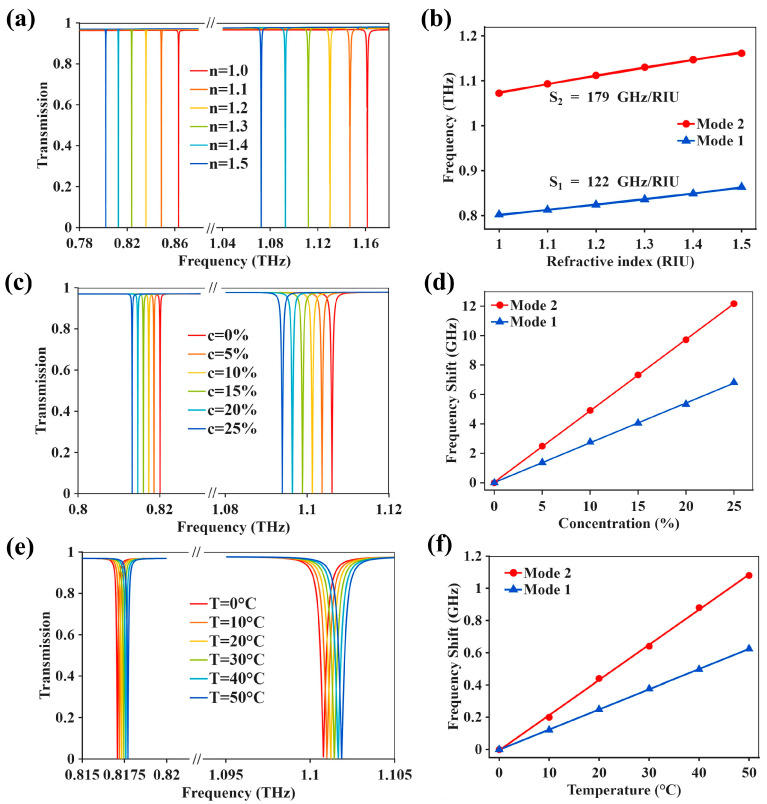
Sensing performance of the metasurface enabled by dual quasi-BIC resonances. (**a**) Transmission spectra for different refractive indices (n = 1.0–1.5). (**b**) Resonance frequencies of Mode 1 and Mode 2 as a function of refractive index. (**c**) Transmission spectra for different CaCl_2_ concentrations (0–25%). (**d**) Frequency shifts of Mode 1 and Mode 2 as a function of concentration. (**e**) Transmission spectra at different temperatures (0–50 °C). (**f**) Frequency shifts of Mode 1 and Mode 2 as a function of temperature.

Because the resonance frequencies and near-field confinement of metasurfaces are strongly influenced by the dielectric loading surrounding the resonators, small variations in local refractive index can be translated into measurable spectral shifts. This perturbation-based mechanism has led to high-Q dielectric metasurfaces, especially quasi-BIC platforms, becoming highly suitable for refractive-index sensing and liquid-phase analyte detection [38,40,41,42,43]. Recent studies have also shown that THz all-dielectric metasurfaces based on BIC or quasi-BIC resonances are able to support dual-resonance readout, improved refractometric sensitivity, and an extended sensing range under appropriately designed symmetry breaking [35,44,45]. In the proposed all-LCP metasurface, the presence of two quasi-BIC resonances in different frequency windows offers two possible spectral observables for refractive-index readout under the same analyte condition. As illustrated in Figure 5a,b, both Mode 1 and Mode 2 show clear monotonic redshifts with increasing refractive index, indicating that the increasing dielectric loading continuously alters the resonance condition of the symmetry-broken tetramer metasurface. Linear fitting gives $f_{1}=0.9832-0.1218n$ and $f_{2}=1.3429-0.1789n$, corresponding to refractive-index sensitivities of 122 GHz/RIU and 179 GHz/RIU for Mode 1 and Mode 2, respectively, with $R^{2}=0.9968$ for both modes, where $R^{2}$ denotes the coefficient of determination of the linear fit. These results indicate that, within the refractive-index range of 1.0–1.5 and under the present structural and analyte-loading conditions, the two absolute resonance frequencies maintain stable linear responses and can therefore serve as dual spectral observables for subsequent sensing analysis.

To relate the refractive-index response to an analyte-relevant parameter mapping, CaCl_2_ solution was selected as a representative model system. According to the empirical relation reported by Tan and Huang [46], the refractive index of CaCl_2_ solution can be expressed as:

(1) $$\;n\approx 1.3339+2.5067\times 10^{-3}c-1.1122\times 10^{-4}\left(T-273.15\right)$$

where c is the concentration in mass percent. This linearized expression directly connects refractive index with concentration and temperature, making it suitable for the present dual-mode sensing framework. Here, the concentration- and temperature-related analysis is established through the empirical refractive-index relation of the analyte model.

Based on this analyte model, concentration sensing was first examined at a fixed temperature of 25 °C. As shown in Figure 5c,d, both resonances exhibit monotonic shifts with CaCl_2_ concentration in the range of 0–25%, and the fitted concentration sensitivities are 270 MHz/% for Mode 1 and 485 MHz/% for Mode 2. Temperature sensing was then evaluated at a fixed concentration of 10%, as illustrated in Figure 5e,f. In this case, the resonance frequencies of Mode 1 and Mode 2 increase about linearly with temperature from 0 to 50 °C, yielding temperature sensitivities of 12.5 MHz/°C and 21.8 MHz/°C, respectively. Although the concentration- and temperature-induced shifts are smaller than the direct refractive-index variations, both perturbations remain spectrally distinguishable by the two quasi-BIC resonances. More importantly, the two modes exhibit different concentration and temperature slopes, indicating that analyte concentration and thermal perturbation are mapped differently onto the two spectral channels [47,48,49,50]. Under the present refractive-index-mapping framework, the dual-mode response therefore provides a basis for concentration- and temperature-related analysis instead of relying only on refractive-index readout.

Accordingly, in the low-refractive-index interval, the frequency shifts of the two modes can be expressed as:

(2) $$\;∆f_{1}=S_{c,1}∆c+S_{T,1}∆T$$

(3) $$\;∆f_{2}=S_{c,2}∆c+S_{T,2}∆T$$

where $∆f_{1}$ and $∆f_{2}$ denote the resonance-frequency shifts of Mode 1 and Mode 2 relative to their corresponding reference states, respectively; ∆c and ∆T represent variations in analyte concentration and temperature; $S_{c,1}$ and $S_{c,2}$ correspond to the concentration sensitivities of Mode 1 and Mode 2; and $S_{T,1}$ and $S_{T,2}$ denote the respective temperature sensitivities. Using the fitted sensitivities extracted from Figure 5, the above relation can be written in matrix form as:

(4) $$\left[\begin{array}{c} ∆f_{1}\\ ∆f_{2} \end{array}\right]=\left[\begin{array}{c} 270\;12.5\\ 485\;21.8 \end{array}\right]\left[\begin{array}{c} ∆c\\ ∆T \end{array}\right]$$

where $∆f_{1}$ and $∆f_{2}$ are in MHz, ∆c is in %, and ∆T is in ℃. In principle, the concentration and temperature perturbations can be obtained through matrix inversion. This formulation is consistent with recent dual-parameter THz metasurface sensing frameworks, in which two spectrally distinguishable resonances are employed to separate coupled analyte variables [51].

**Figure 6:**
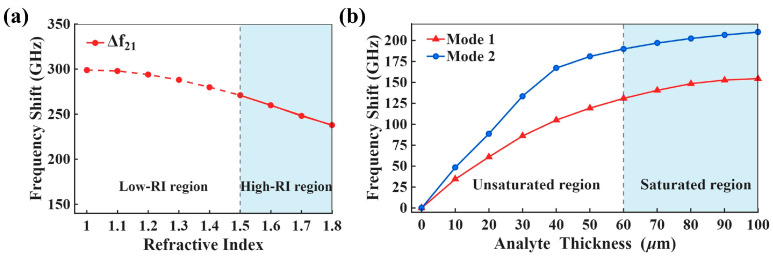
(**a**) Frequency difference between the two resonant modes ($∆f_{21}$) as a function of refractive index. (**b**) Frequency shifts of Mode 1 and Mode 2 versus analyte thickness at n = 1.5.

Figure 5 therefore indicates that, under the present structural parameters and analyte-loading conditions, both quasi-BIC modes provide stable and nearly linear absolute-frequency responses in the low-index regime. However, this advantage does not remain equally effective over the entire refractive-index range. When the refractive index is further increased, the individual resonance frequencies gradually exhibit reduced linear consistency compared with their behavior in the 1.0–1.5 interval, which makes it necessary to consider alternative spectral observables. In this context, Figure 6a presents the intermodal frequency difference:

(5) $$∆f_{21}=f_{2}-f_{1}$$

as a function of refractive index, where $f_{1}$ and $f_{2}$ are the absolute resonance frequencies of Mode 1 and Mode 2, respectively. Over the full range of 1.0–1.8, $∆f_{21}$ does not maintain the same level of linearity as the absolute resonance frequencies in the low-index interval. By contrast, when the refractive index enters the interval of 1.5–1.8, $∆f_{21}$ becomes highly linear and can be fitted by $∆f_{21}=437.4-111n$, where $∆f_{21}$ is in GHz and n is the refractive index. This result suggests that the present metasensor is more suitably described by a regime-dependent readout strategy rather than a single spectral observable across the entire refractive-index range. Specifically, in the low-index regime (1.0–1.5), the absolute resonance frequencies $f_{1}$ and $f_{2}$ act as the primary observables because both maintain stable linear responses and can further support concentration- and temperature-related analysis. In the higher-index regime (1.5–1.8), the intermodal frequency difference $∆f_{21}$ becomes the preferred observable because it exhibits improved linearity under the current design conditions. It should be noted that this transition boundary is determined empirically for the present structure and analyte-loading model, rather than being presented as a universal threshold.

The advantage of the differential quantity can be further understood from its perturbation form:

(6) $$\;\delta \left(∆f_{21}\right)=\left(S_{x,2}-S_{x,1}\right)\delta x+\left(S_{p,2}-S_{p,1}\right)\delta p$$

where $S_{x,1}$ and $S_{x,2}$ represent the sensitivities of Mode 1 and Mode 2 to the target variable x, $S_{p,1}$ and $S_{p,2}$ denote the sensitivities to the parasitic perturbation p, and δx and δp correspond to the respective perturbation magnitudes. When $S_{p,2}$ and $S_{p,1}$ are close to each other, the parasitic term is reduced in the differential signal, whereas the contribution from the target variable can still be retained through the sensitivity difference $\left(S_{x,2}-S_{x,1}\right)$. Therefore, compared with tracking the two absolute resonance frequencies separately, differential readout is expected to be less sensitive to common-mode perturbations within the present numerical framework, particularly in the higher-index regime.

The dependence of analyte response on thickness is further examined in Figure 6b at a fixed refractive index of n = 1.5. As the analyte thickness increases from 0 to 100 μm, the resonance shifts of both modes show a rapid increase at the initial stage and then gradually tend to saturation. In this context, the analyte thickness is represented by t, and the resonance shift is defined with respect to the thickness-free condition (t = 0). By taking this condition as the reference, the total shifts at t = 100 μm reach 154.53 GHz for Mode 1 and 210.05 GHz for Mode 2, respectively. These results indicate a pronounced thickness-dependent near-field response of the proposed all-LCP metasurface, demonstrating its sensitivity to analyte loading variations near the surface. The thickness range considered here helps reveal the effective sensing depth of the metasurface through the gradual evolution of the spectral response.

## 4. Conclusions

In conclusion, this work presents an LCP THz metasensor platform with dual quasi-BIC resonances for dual-range refractive-index sensing. By introducing structural asymmetry into a periodic LCP tetramer metasurface, two high-Q resonances were generated at 0.863 THz and 1.162 THz, with Q factors of 6811 and 2526, respectively. In the low-refractive-index regime of 1.0–1.5, the two resonance frequencies exhibit stable linear responses, with sensitivities of 122 GHz/RIU and 179 GHz/RIU, thereby providing dual spectral channels for refractive-index readout. By integrating the refractive-index response with the refractive-index–concentration–temperature relation of CaCl_2_ solution, the proposed metasurface is further extended to concentration- and temperature-dependent sensing, thereby providing a basis for dual-mode multiparameter analysis. In the higher refractive-index range of 1.5–1.8, the intermodal frequency difference $∆f_{21}$ exhibits improved linearity and is considered a more stable differential observable compared to the absolute resonance frequencies. The importance of this work is reflected not only in the realization of high-Q dual-mode THz sensing, but also in the establishment of a regime-dependent readout strategy for metasurface sensors. Rather than applying a single spectral observable across the entire sensing range, a more suitable observable is selected according to the dielectric-response regime, which enhances sensing adaptability and readout reliability. Together with the material benefits of LCP, such as low loss, simple structure, and compatibility with scalable fabrication, this approach offers a practical route toward THz biochemical sensing devices. Further extension to realistic aqueous biosensing will require additional assessment under lossy liquid conditions, where water absorption may affect resonance visibility and readout performance.

## Figures and Tables

**Table 1:** Comparison of representative all-dielectric THz sensing platforms relevant to the present work.

Ref.	Structure	Operating Band	Q Factor	Sensitivity	FOM
[20]	LiTaO_3_ tetramer clusters	2.41 THz	—	135 GHz/RIU	—
[35]	Silicon tetramer clusters	2.397/2.442 THz	2303.77	46/67 GHz/RIU	7022.9/4373.4
[36]	Silicon asymmetric a-SRRs	~0.78 THz	~75.7	231 GHz/RIU	~12.7
[39]	PDMS stretchable cube clusters	~0.96 THz	872	217 GHz/RIU	197
This work	LCP tetramer clusters	0.863/1.162 THz	6811/2526	122/179 GHz/RIU	963.1/389.1

## Data Availability

The raw data supporting the conclusions of this article will be made available by the authors on request.
